# Functional Analysis of Sterol O-Acyltransferase Involved in the Biosynthetic Pathway of Pachymic Acid in *Wolfiporia cocos*

**DOI:** 10.3390/molecules27010143

**Published:** 2021-12-27

**Authors:** Wenjun Zhu, Ying Liu, Jing Tang, Heping Liu, Naliang Jing, Fengfeng Li, Ran Xu, Shaohua Shu

**Affiliations:** 1 School of Life Science and Technology, Wuhan Polytechnic University, Wuhan 430023, China; zhuwenjun2002@163.com; 2 College of Plant Science and Technology, Huazhong Agricultural University, Wuhan 430070, China; 18186421026@163.com (Y.L.); hzautj@163.com (J.T.); liuheping1236@163.com (H.L.); jnl_1997@163.com (N.J.); lifengfeng@mail.hzau.edu.cn (F.L.)

**Keywords:** pachymic acid, biosynthetic pathway, *WcSOAT*, genes modification, triterpenoids

## Abstract

Pachymic acid from *Wolfiporia cocos* possesses important medicinal values including anti-bacterial, anti-inflammatory, anti-viral, invigorating, anti-rejection, anti-tumor, and antioxidant activities. However, little is known about the biosynthetic pathway from lanostane to pachymic acid. In particular, the associated genes in the biosynthetic pathway have not been characterized, which limits the high-efficiency obtaining and application of pachymic acid. To characterize the synthetic pathway and genes involved in pachymic acid synthesis, in this study, we identified 11 triterpenoids in *W. cocos* using liquid chromatography tandem mass spectrometry (LC-MS/MS), and inferred the putative biosynthetic pathway from lanostane to pachymic acid based on analyzing the chemical structure of triterpenoids and the transcriptome data. In addition, we identified a key gene in the biosynthetic pathway encoding *W. cocos* sterol O-acyltransferase (*WcSOAT*), which catalyzes tumolusic acid to pachymic acid. The results show that silence of *WcSOAT* gene in *W. cocos* strain led to reduction of pachymic acid production, whereas overexpression of this gene increased pachymic acid production, indicating that *WcSOAT* is involved in pachymic acid synthesis in *W. cocos* and the biosynthesis of *W. cocos* pachymic acid is closely dependent on the expression of *WcSOAT* gene. In summary, the biosynthetic pathway of pachymic acid and the associated genes complement our knowledge on the biosynthesis of *W. cocos* pachymic acid and other triterpenoids, and also provides a reference for target genes modification for exploring high-efficiency obtaining of active components.

## 1. Introduction

The sclerotia of brown rot fungus *Wolfiporia cocos*, also named fuling in China, possess important edible and medicinal values that have been used as both edible mushroom and traditional medicine in East Asia for centuries [1]. The edible *W. cocos* sclerotium has been called “Indian bread” or “tuckahoe” in North America [2]. Furthermore, fuling has been used as a crude drug as a stomachic, diuretic, and sedative in traditional Chinese and Japanese herbal medicine [3,4]. Fuling is also widely used as a supplement in multiple wines, nutraceuticals, functional foods, cosmetics, and tea to improve the quality [5,6,7].

Polysaccharides and triterpenoids are the major bioactive components of *W. cocos* sclerotia. Recently, several studies reported that the polysaccharides and triterpenoids possess multiple immune-stimulatory activities, anti-bacterial, anti-inflammatory, anti-viral, invigorating, anti-rejection, anti-tumor, and antioxidant activities [8,9,10,11,12,13,14]. Pachymic acid isolated from *W. cocos* is a triterpenoid, which belongs to the derivative of lanostane skeleton [2,15,16]. Several studies have reported that pachymic acid is one of the major pharmacologically active compounds from *W. cocos* with anti-inflammatory and anti-tumor activities [10,17].

Development and cultivation of fuling, the sclerotia of *W. cocos*, are both dependent on colonization on the wood of *Pinus* species [18,19]. In China, artificially cultivated fuling can reach an average weight of 3–4 kg [19]. Currently, the commercial production of fuling is limited because of severe destruction of *W. cocos* habitat and the shortages in pinewood resources. Furthermore, the quantity and quality of active constituents in fuling vary broadly because of the diverse *W. cocos* cultivars, cultivation conditions, and pine woods from different regions of China. All these cause difficulty for standardized production and clinical application of *W. cocos* preparations. However, the *W. cocos* inheritance mechanism and the genetic basis of sclerotial development and active compound formation are still largely unknown. Based on optimized biosynthetic pathways, synthetic biology has used metabolic-pathway design and genetic elements to develop organisms that can synthesize important chemicals [20]. Synthetic biology technology has been successfully used for the synthesis of many secondary metabolites and bioactive compounds [21,22,23,24]. Therefore, the synthesis of fuling active compounds using synthetic biology technology was a viable and efficient strategy that could stably produce active compounds and reduce the consumption of pinewood resources.

Elucidating the biosynthetic pathway and the associated catalyticase genes are key steps to produce target compounds, heterogeneously or homologously. At present, although the synthesis of lanostane, the important precursor of pachymic acid, via mevalonic acid (MVA) pathway has been well studied [25,26], little is known about the biosynthetic pathway from lanostane to pachymic acid in *W. cocos*. Identification of the associated genes remains a challenge.

To better understand the biosynthetic mechanism, in this study, we identified 11 triterpenoids in *W. cocos* using liquid chromatography-mass spectrometry/mass spectrometry (LC-MS/MS) and analyzed their chemical structure. Furthermore, by analyzing the transcriptome data from our previous study [26], we also inferred the potential biosynthetic pathway from lanostane to pachymic acid. In addition, we identified a key gene encoding *W. cocos* sterol O-acyltransferase (*WcSOAT*), which catalyzes tumolusic acid to pachymic acid. SOAT is conserved in eukaryote that catalyzes sterol esterification to detoxify [27], but its function in *W. cocos* is still unknown.

To elucidate the pachymic acid synthetic pathway and characterize the *WcSOAT* gene in pachymic acid synthesis, we silenced and overexpressed the *WcSOAT* gene in *W. cocos.* The results indicate that WcSOAT plays a significant role. Functional characterization of WcSOAT will help reveal the synthesis pathway of pachymic acid in *W. cocos* and ways for producing the active components efficiently.

## 2. Results

### 2.1. Determination of Triterpenoids Compounds in W. cocos

Total triterpenoids in *W. cocos* mycelium and sclerotia were determined. In total, 11 triterpenoids were identified by HPLC–MS qualitative analysis: pachymic acid, eburicoic acid, lanosterol, trametenolic acid, tumulosic acid, dehydrotrametenolic acid, 16-α-hydroxytrametenolic acid, poricoic acid C, 3-epi-dehydrotumulosic acid, 3-epi-dehydropachymic acid, and dehydroeburicoic acid (Figure S1).

### 2.2. Biosynthetic Pathway Inferring and Annotation of Catalytic Enzymes Genes

**Figure 1:**
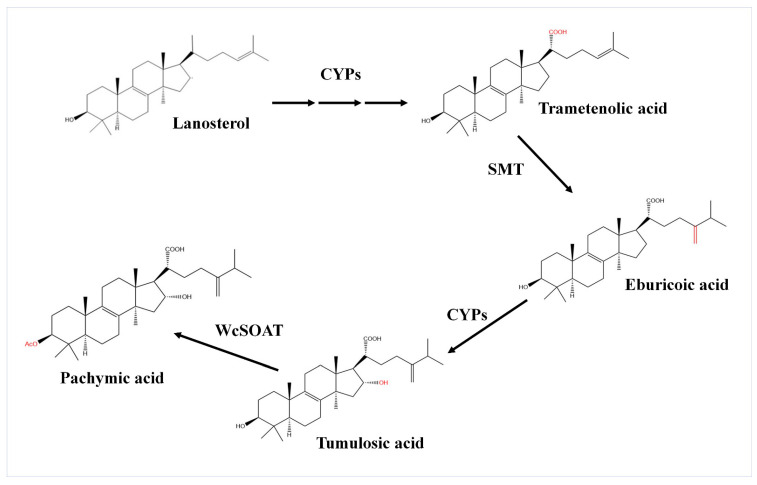
The putative biosynthetic pathway from lanostane to pachymic acid in *W. cocos.* CYPs: cytochrome P450 family protein; SMT: sterol C-24 methyltransferase; WcSOAT: *W. cocos* sterol O-acyltransferase.

*G. lucidum* and *W. cocos* may share a highly similar biosynthesis pathway. Therefore, based on the chemical structure and chemical groups of identified triterpenoids, and according to the biosynthetic pathway of ganoderic acid, the possible biosynthetic pathway from lanostane to pachymic acid was inferred. (1) The C (21) of lanosterol is oxidized by cytochrome P450 family protein (CYPs) to generate carboxyl and form trametenolic acid. (2) The C (24) of trametenolic acid is catalyzed by sterol C-24 methyltransferase (SMT) to form eburicoic acid. (3) The C (16) of eburicoic acid is oxidized by cytochrome P450 family protein to form tumulosic acid. (4) The C (3) of tumulosic acid is acetylized by sterol O-acetyl transferase (SOAT) to form pachymic acid (Figure 1).

In addition, according to the transcriptome data of *W. cocos* [26], 128 unigenes encoding cytochrome P450s, 2 unigenes encoding SMTs, and 9 unigenes encoding SOATs were mined. BLAST searches of *KEGG* and *NCBI* database with annotated SOATs sequence revealed the cDNA sequence of *W. cocos* sterol O-acyltransferase (*WcSOAT*) gene (NCBI ID: KY800894.1).

### 2.3. WcSOAT Is a Transmembrane Sterol O-Acyltransferase

**Figure 2:**
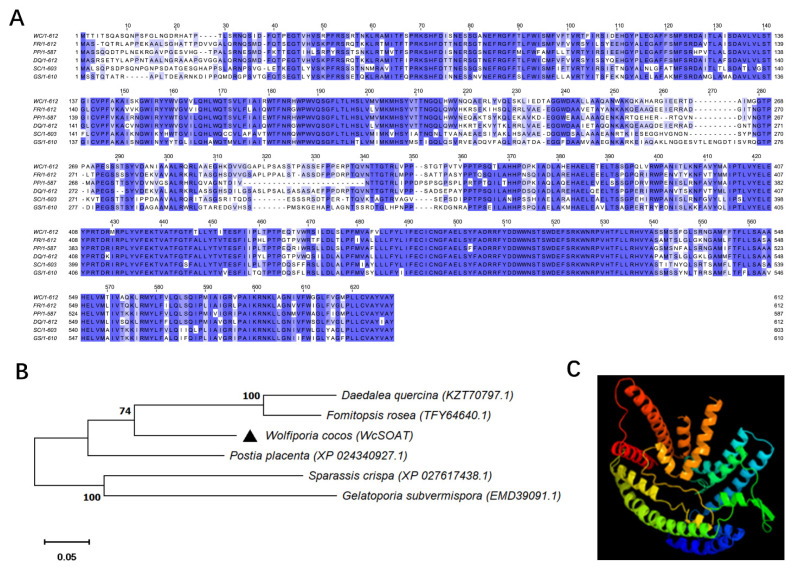
Sequence similarities between WcSOAT and its homologs. (**A**) Multiple sequence alignment of WcSOAT and its homologs. Full-length protein sequences were aligned using Clustal W and the alignment was edited using Jalview. Intensity of blue shading reflects the level of amino acid identity at each position. WC: *W. cocos*; FR: *Fomitopsis rosea* (TFY64640.1, 77.51%); PP: *Postia placenta* (XP_024340927.1, 75.25%); DQ: *Daedalea quercina* (KZT70797.1, 76.25%); SC: *Sparassis crispa* (XP_027617438.1, 65.95%); GS: *Gelatoporia subvermispora* (EMD39091.1, 63.92%). (**B**) Phylogenetic analysis of WcSOAT and its homologs from other species. The full-length protein sequences were analyzed using MEGA X with Unrooted neighbor-joining bootstrap (1000 replicates). The black triangle marks the location of WcSOAT. A scale bar at the lower left corresponds to a genetic distance of 0.05. (**C**) 3D structural models of WcSOAT, predicted using I-TASSER and further analyzed by PyMOL software.

The *WcSOAT* gene encodes a protein of 612 amino acids. No signal peptide was predicted using SignalP 5.0 server. Seven transmembrane helices were predicted using TMHMM Server version 2.0, indicating that the protein is a transmembrane protein. The ProtComp 9.0 analysis indicated that the sub-cellular localization of WcSOAT protein is on the endoplasmic reticulum. The SMART MODE analysis showed that WcSOAT contains a MBOAT (membrane bound O-acyltransferase) domain (Pfam ID: PF03062) with the function of acetylating the hydroxyl group. BLAST searches of WcSOAT sequence against the NCBI database revealed the high similarity homologs of WcSOAT in a large number of fungal genera. Multiple sequence alignment and phylogenetic analysis of WcSOAT and its homologues showed significant sequence similarity (Figure 2A,B). The 3D structure of WcSOAT predicted by *Phyre2* showed that WcSOAT mainly consists of α-helix (Figure 2C), indicating that WcSOAT is a hydrophobin. In sum, these results indicated that WcSOAT functions as a transmembrane sterol O-acyltransferase.

### 2.4. MeJA Induces WcSOAT Gene Expression and Pachymic Acid Accumulation in W. cocos

**Figure 3:**
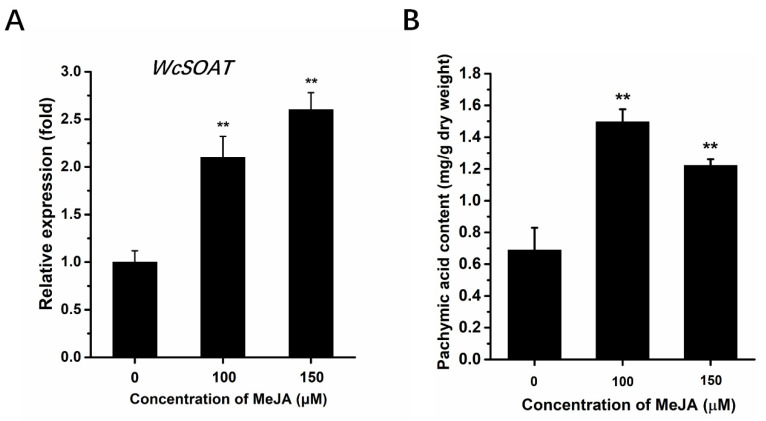
The effects of MeJA on *WcSOAT* gene expression and pachymic acid accumulation. (**A**) The relative expression level of *WcSOAT* in *W. cocos* after treatment with MeJA was evaluated by RT-qPCR. The expression level of *WcSOAT* in the sample treated without MeJA was set as 1, and relative transcript levels were calculated using the comparative Ct method. Transcript levels of the *W. cocos his3-1* gene were used to normalize different samples. Data represent means and standard deviations of three independent replications. (**B**) The content of pachymic acid in *W. cocos* after treatment with MeJA. Data were obtained from three independent experiments with total 9 replicates. ** indicate statistical differences at *p* ≤ 0.01 compared with sample without MeJA treatment, one-way ANOVA.

The effects of MeJA on the genes involved in triterpenoids biosynthesis have been reported in plants and fungi [28,29,30,31]. Therefore, we infer that MeJA may regulate the expression of *WcSOAT* gene. The results showed that the expression levels of *WcSOAT* increased significantly after MeJA treatment (Figure 3A), indicating that MeJA can induce the *WcSOAT* gene expression.

Several studies have shown that MeJA functions as an enhancer of pachymic acid accumulation by activating the triterpenoids biosynthesis pathway [29,31]. We analyzed the effect of MeJA on the accumulation of pachymic acid using the HPLC method. This method in our study showed a good linear relationship (Figure S2), and is accurate, reproducible, and stable, and can be used to determine the content of pachymic acid in *W. cocos.* The results showed that the levels of pachymic acid accumulation in *W. cocos* mycelium increased significantly after MeJA treatment (Figure 3B), suggesting that MeJA may induce the accumulation of pachymic acid by increasing the supply of precursors.

### 2.5. WcSOAT Is Involved in Pachymic Acid Synthesis

**Figure 4:**
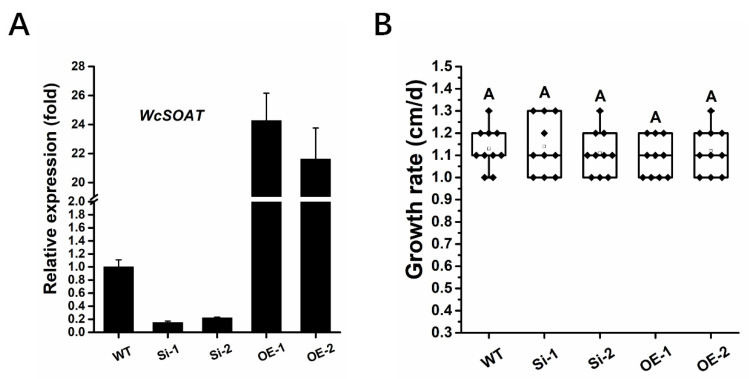
The phenotype of *WcSOAT* gene silence and overexpression strains. (**A**) The relative expression level of *WcSOAT* in WT, *W. cocos* gene silence (Si), and overexpression (OE) strains. The expression level in WT strain was set as 1, and relative transcript levels were calculated using the comparative Ct method. Transcript levels of the *W. cocos his3-1* gene were used to normalize different samples. Data represent means and standard deviations of three independent replications. (**B**) Hyphal growth rate. The indicated strains were grown on PDA plates at 25 °C. Radial growth was measured every day for 5 days and growth rate was calculated. Data were obtained from three independent experiments with total 10 replicates. In box plots, whiskers indicate the minimum and maximum values; the line indicates the median, the box boundaries indicate the upper (25th percentile) and lower (75th percentile) quartiles, all data are plotted as black dots. Same letters in the graph indicate no statistical differences at *p* ≤ 0.01 using one-way ANOVA one-way.

To determine the possible functions of *WcSOAT* in the synthesis of pachymic acid, we generated *WcSOAT* gene silence and overexpression strains, and the expression levels of the *WcSOAT* gene in wild type (WT), silence (Si), and overexpression (OE) strains were verified using RT-qPCR (Figure 4A).

**Figure 5:**
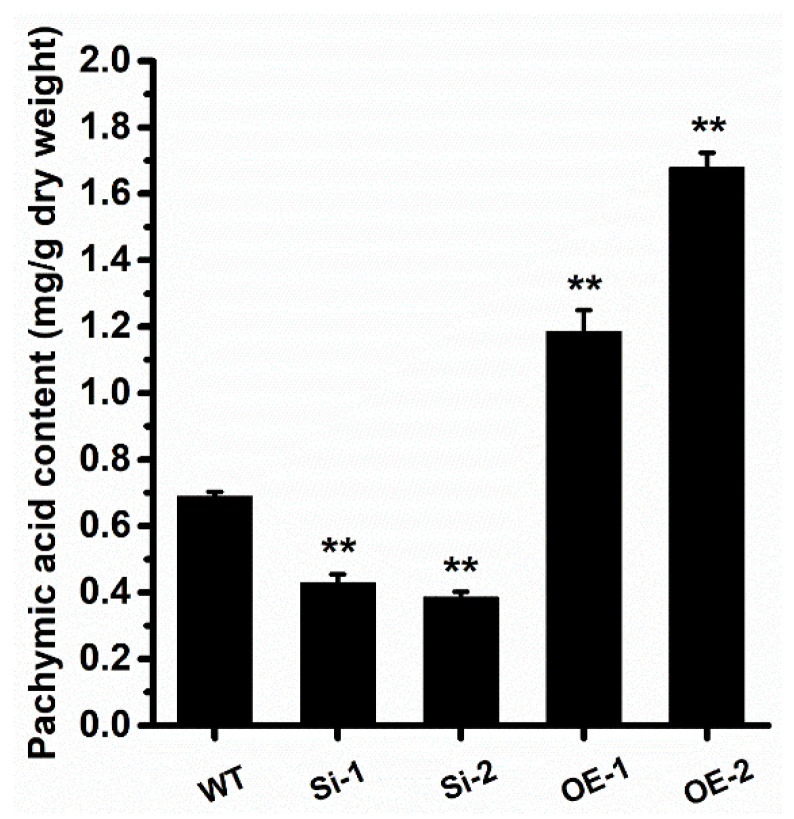
*WcSOAT* is involved in pachymic acid synthesis. The content of pachymic acid in indicated *W. cocos* strains was analyzed. Data were obtained from three independent experiments with total 9 replicates. ** indicate statistical differences at *p* ≤ 0.01 compared with WT strain, one-way ANOVA.

We then analyzed whether the *WcSOAT* gene affects the growth of *W. cocos.* The result showed no difference in growth on PDA in the gene silence and overexpression strains compared with the wild-type strain (Figure 4B). Then, we analyzed the concentration of pachymic acid. The results demonstrated that the production of pachymic acid decreased in *WcSOAT* gene silence strains, whereas the production increased in *WcSOAT* overexpression strains, compared with wild-type strain (Figure 5), indicating that WcSOAT is involved in pachymic acid synthesis.

## 3. Discussion

Pachymic acid, a triterpenoid from traditional Chinese medicinal fungus *W. cocos*, possesses pharmacological activities, including anti-inflammatory and anti-tumor activities [10,17]. However, compared with the well-studied lanostane synthesis pathway [25,26], the biosynthetic pathway from lanostane to pachymic acid remains incompletely understood. Furthermore, the functions of the genes involved in biosynthesis of pachymic acid remain only partly known, limiting the genetic manipulation for enhancing the production of pachymic acid.

Previous studies on *Ganoderma lucidum*, the closely related fungi of *W. cocos*, revealed the biosynthetic pathway from lanostane to ganoderic acid. The lanosterol is catalyzed by cytochrome P450 protein CYP5150L8 to form 3-hydroxy-lanosta-8,24-dien-26-ol (HLDO), then form 3-hydroxy-lanosta-8,24-dien-26-al (HLDA), and finally form lanosterol derivative ganoderic acid [32,33]. Pachymic acid and ganoderic acid are both derivatives of lanosterols and share a highly similar carbon skeleton with other lanosterol derivatives. *G. lucidum* and *W. cocos* may also share a highly similar biosynthesis pathway. Therefore, we firstly inferred the putative biosynthetic pathway from lanostane to pachymic acid and annotated the catalyticase genes based on the triterpenoids structure and transcriptome data of *W. cocos*, and the biosynthetic pathway of ganoderic acid (Figure 1). The results demonstrated that the C (21) of lanosterol is oxidized by cytochrome P450 family protein to generate carboxyl and form trametenolic acid, then the C (24) of trametenolic acid is catalyzed by SMT to form eburicoic acid, the C (16) of eburicoic acid is oxidized by cytochrome P450 family protein to form tumulosic acid, and the C (3) of tumulosic acid is acetylized by SOAT to form pachymic acid (Figure 1).

In this study, 11 chemical structures of triterpenoids were identified in *W. cocos* using HPLC–MS qualitative analysis (Figure S1). These provided the basis for predicting the biosynthetic pathway of pachymic acid in *W. cocos*. For further gene function annotation, we employed the newest biological informatics, including genome and transcriptome data of *W. cocos*, to annotate the genes in the biosynthetic pathway, and then inferred a more comprehensive synthesis pathway of pachymic acid in *W. cocos*. In this pathway, WcSOAT catalyzes the last step in pachymic acid synthesis pathway: acetylizing the C (3) of tumulosic acid to form pachymic acid (Figure 1). In *Arabidopsis thaliana*, the sterol O-acyltransferase AtSAT1 mediates phytosterol ester biosynthesis [34]. However, the functions of SOAT in the synthesis of pharmacologically active compounds are still largely unknown in medicinal fungi. Furthermore, although the genome [25,35], transcriptome [26], secretome [36], and mitochondrial genome [37] of *W. cocos* have been investigated, the molecular mechanisms involved in the biosynthesis of secondary metabolites remained incompletely understood. The gene function study of *WcSOAT* has not been reported before. For the first time, we analyzed the function of *WcSOAT* gene in pachymic acid synthesis. The results revealed that genetic manipulation of the expression level of *WcSOAT* gene could change the pachymic acid production (Figure 5), indicating that *WcSOAT* is involved in biosynthesis of pachymic acid in *W. cocos*. Therefore, gene function study will provide novel resources for investigating the mechanisms driving the synthesis of active compounds in *W. cocos*, and more genes need to be investigated in further study.

Diverse environmental signals can regulate the expression of genes related to the biosynthesis of secondary metabolites [38,39,40]. MeJA is an important signaling component in plants and fungi. Our result showed that exogenous MeJA could induce the *WcSOAT* gene expression and enhance the pachymic acid accumulation in *W. cocos* (Figure 3). This result provides another clue that the signaling components can be applied as inducers to increase the accumulation of pharmacologically active secondary metabolites in medicinal plants or fungi, and function study on the synthesis of the active compounds associated genes will also help elucidate the regulatory mechanisms of active compounds in *W. cocos* and other fungi.

In summary, we determined the triterpenoids compounds, inferred the biosynthetic pathway from lanostane to pachymic acid, and analyzed the functions of *WcSOAT* gene in pachymic acid synthesis in *W. cocos*. The results of this study will help reveal the synthesis pathway of pachymic acid in *W. cocos* and produce the active components more efficiently and will also provide new insights into the molecular mechanisms involved in secondary metabolism in *W. cocos*.

## 4. Materials and Methods

### 4.1. Strains and Culture Conditions

The wild type *W. cocos* strain GIM5.219 was obtained from the Institute of Agricultural Culture Collection of China and used to generate gene silence and gene over expression transformants in this study. All the strains in this study were grown on potato dextrose agar (PDA) medium (Acumedia, Lansing, MI, USA) at 25 °C. *Escherichia coli* strain JM109 was used for propagating all plasmids and grown on Luria–Bertani (LB) medium (Difco, Detroit, MI, USA) containing 100 μg/mL ampicillin or 50 μg/mL kanamycin as required at 37 °C.

### 4.2. Bioinformatics Analysis and Programs Used in This Study

The SignalP 5.0 server (http://www.cbs.dtu.dk/services/SignalP/ accessed on 8 October 2021) was used to predict the signal peptide sequence and SMART MODE (http://smart.embl-heidelberg.de/smart/change_mode.pl accessed on 8 October 2021) was used to analyze the protein domain. The 3D structural model of WcSOAT was analyzed using I-TASSER (http://zhanglab.ccmb.med.umich.edu/I-TASSER/accessed on 26 October 2021). The National Center for Biotechnology Information (NCBI) (http://www.ncbi.nlm.nih.gov/ accessed on 26 October 2021) and Kyoto Encyclopedia of Genes and Genomes (KEGG) (https://www.genome.jp/kegg/accessed on 26 October 2021) databases were used for BLAST analysis. The ProtComp 9.0 (http://www.softberry.com/berry.phtml?topic=protcompan&group=programs&subgroup=proloc accessed on 26 October 2021) was used for sub-cellular localization prediction of WcSOAT protein. The TMHMM Server v. 2.0 (http://www.cbs.dtu.dk/services/TMHMM/accessed on 26 October 2021) was used for transmembrane structure prediction.

### 4.3. Extraction and Manipulation of DNA and RNA

Genomic DNA of indicated *W. cocos* strains were isolated using the CTAB method as described previously [41]. The total RNA of indicated strains were isolated employing TriZOL reagent (Invitrogen, Waltham, MA, USA) according to the manufacturer’s protocols. The total RNA samples were treated with DNase I (Thermo Scientific, Vilnius, Lithuania) to digest the residual genomic DNA, and then used to generate the first strand cDNA using RevertAid^TM^ First Strand cDNA Synthesis Kit (Thermo Scientific, Vilnius, Lithuania).

The RT-qPCR was used to determine the gene expression levels of *WcSOAT* gene in indicated strains. RT-qPCR was performed using the CFX96 Touch Real-Time PCR Detection System (Bio-Rad, Hercules, CA, USA) and SYBR Premix Ex Taq II (Takara, Dalian, China), following the manufacturer’s instructions. Primers were designed across or flanking an intron (WcSOAT-Q-F: ATCTCCGATGCTGTGCTCGTTCT and WcSOAT-Q-R: CAGCGTAAGGAAGCCCGAC). The relative expression levels of the *W. cocos his3-1* gene was used as references for normalizing the RNA sample (his3-1-F: AGTCCACGGAACTCCTAATCA and his3-1-R: AGCGGCTAAGTTGGTGTCT). For each examined gene, RT-qPCR assays were repeated three times, each repetition with three independent replicates.

To verify the effect of MeJA on the expression of *WcSOAT* gene, wild type *W. cocos* strain was cultured on PDA or PDA containing 100 μM MeJA at 28 °C for 5 days, then the expression level of *WcSOAT* was analyzed using RT-qPCR.

### 4.4. Plasmid Construction and Transformation of W. cocos

To construct the *WcSOAT* gene over expression vector, the full-length open reading frame of *WcSOAT* gene fused with HA tag at the C terminus was amplified by PCR with restriction sites of *Asc* I and *Not* I, and then ligated to plasmid pHygKS under the manipulation of *Ganoderma lucidium glyceraldehyde-3-phosphate dehydrogenase* gene promoter (*Pgpd*) (NCBI Accession: DQ404345.1) and the endo-b-1,4-glucanase precursor terminator (NCBI Accession: CP009807.1).

To construct the *WcSOAT* gene silence vector, a 400 bp fragment was PCR amplified from *WcSOAT* cDNA sequence with indicated restriction sites and then ligated to pCIT to construct a reverse repeat structure that separated by the 420 bp intron. This new reverse repeat fragment was digested with *Asc* I and *Not* I, then ligated with the pHygKS plasmid to generate the *WcSOAT* gene silence vector.

Transformation of *W. cocos* was performed as described previously [42].

### 4.5. Chemical Analysis Methods

#### 4.5.1. Fungus Material Preparation

The *W. cocos* sclerotium were milled into a powder and oven dried at 25 °C until the weight remained constant. Then, 1 g powdered samples were subjected to soaking in 10 mL methanol and ultrasound lysis (500 W, 40 kHz: 60 min). The mixture was precisely weighed and heated to reflux for 30 min, cooled, and then centrifuged at 9000× *g* for 10 min. The upper layer was collected and filtered through a 0.45 µm filter before using. The standard substance (minimum content: 98%, Batch No. 031006) was provided by Yeyuan Biological company (Shanghai, China).

#### 4.5.2. HPLC-MS Conditions

HPLC–MS analysis was performed using a HPLC system (Agilent Technologies, Santa Clara, CA, USA) coupled to an electrospray ionization–QTOF/MS apparatus (Q-Exactive, Thermo, Waltham, MA, USA). A C18 reversed phase column (Hypersil BDS C18, 250 × 4.6 mm, 5 μm inner diameter, ThermoFisher Scientific, Shanghai, China) was used for HPLC separation. The column temperature was kept at 30 °C, and the flow rate was maintained at 0.8 mL/min. The gradient was composed of acetonitrile (A) and 0.1% phosphoric acid in ultrapure water (B). The linear gradient was set as follows: 0–20 min, 0–50% A; 20–30 min, 50–65% A; 30–51 min, 65–70% A; 51–70 min, 70–85% A; 70–76 min, 85–95% A; 76–85 min, 95–100% A. The extracted samples were used for quantification at a wavelength of 210 nm and with 1.0 μL injection volume.

Target compounds were identified using HPLC-MS/MS as described by Zou et al. [43]. A nitrogen drying gas flow of 600 L·h^−1^, a desolvation temperature at 350 °C, a nebulizer pressure of 45 psi, and a capillary voltage of 2600 V were used. Argon was used as collision energy of 30 V. The MS data were collected in centroid mode from *m*/*z* 200 to 1300 to ensure accurate mass measurements over a wide dynamic range. Dynamic range enhancement was applied throughout the MS experiment. For the precursor ions and fragment, the accurate mass and composition ions were calculated using Mass Lynx V4.1 software (Waters Corporation) included with the instrument. The ESI interface was operated in the positive and negative ion modes.

#### 4.5.3. HPLC Conditions

Agilent 1260 Infinity II HPLC system was used for the analysis. In addition, analysis was carried out on a Hypersil BDS C18 (5 μm, 250 mm × 4.6 mm, internal diameter, ThermoFisher Scientific, China) with a gradient elution program. The mobile phase was composed of acetonitrile (A) and 0.1% phosphoric acid in ultrapure water (B). The elution program was optimized and conducted as follows. The process of separation was performed using the following elution gradient: 0–20 min, 0–50% A; 20–30 min, 50–65% A; 30–51 min, 65–70% A; 51–70 min, 70–85% A; 70–76 min, 85–95% A; 76–85 min, 95–100% A, at a flow rate of 0.8 mL/min. The column temperature was maintained at 30 °C. The external standards and extracted samples were used for quantification at wavelength of 210 nm and with 20 μL injection volume.

#### 4.5.4. Precision, Repeatability and Accuracy

Intra- and inter-day variations were chosen to determine the precision of the developed method. The intra-day variation was determined by analyzing the same mixed standard solution six times within one day, while for the inter-day variability test, the solution was examined in triplicate for consecutive 24 h. The RSD of the retention time and peak area were taken as the measures of precision. The RSD was taken as the measure of repeatability. A recovery test was used to evaluate the accuracy of this method.

### 4.6. Function Annotation of Catalytic Enzymes Genes in the Biosynthetic Pathway of Pachymic Acid

The transcriptome analyses of mycelium and sclerotia of *W. cocos* were performed as described [26]. De novo transcriptome assembly was carried out with the Trinity short reads assembling program. A total of 38,722,186 reads of mycelium and 39,710,244 sclerotium were obtained by further sequence stitching and de-redundancy treatment using TGICL software. BLASTX alignments of all unigenes sequences against several protein databases, including NR, NT, Swiss-Prot, GO, KEGG, and other databases, were performed, with a total of 27,325 unigenes annotations. The candidate genes were screened by analyzing the data of *W. cocos* transcriptome and the differentially expressed genes (DEGs) between mycelium and sclerotia, and the possible genes are selected by blasting against the GO database, KEGG transcription database, and NCBI database.

## Data Availability

Publicly available datasets were analyzed in this study. This data can be found in NCBI (https://www.ncbi.nlm.nih.gov/, accessed on 26 November 2021). The NCBI IDs of genes used in Figure 2 are: KY800894.1, TFY64640.1, XP_024340927.1, KZT70797.1, XP_027617438.1 and EMD39091.1.

## Supplementary Materials

The following supporting information can be downloaded at. Figure S1. Total triterpenoids identified in *W. cocos* mycelium and sclerotia by HPLC–MS qualitative analysis. Figure S2. Standard curve of pachymic acid.
