# Supplementary material for: Functional Analysis of Sterol O-Acyltransferase Involved in the Biosynthetic Pathway of Pachymic Acid in Wolfiporia cocos
Source: Molecules. 2021 Dec 27;27(1):143. doi: 10.3390/molecules27010143 (PMC8746942; doi:10.3390/molecules27010143)

Supplemental Material

Figure S1. Total triterpenoids identified in *W. cocos* mycelium and sclerotia by HPLC–MS qualitative analysis.

Pachymic acid ($C_{33}H_{52}O_5$)

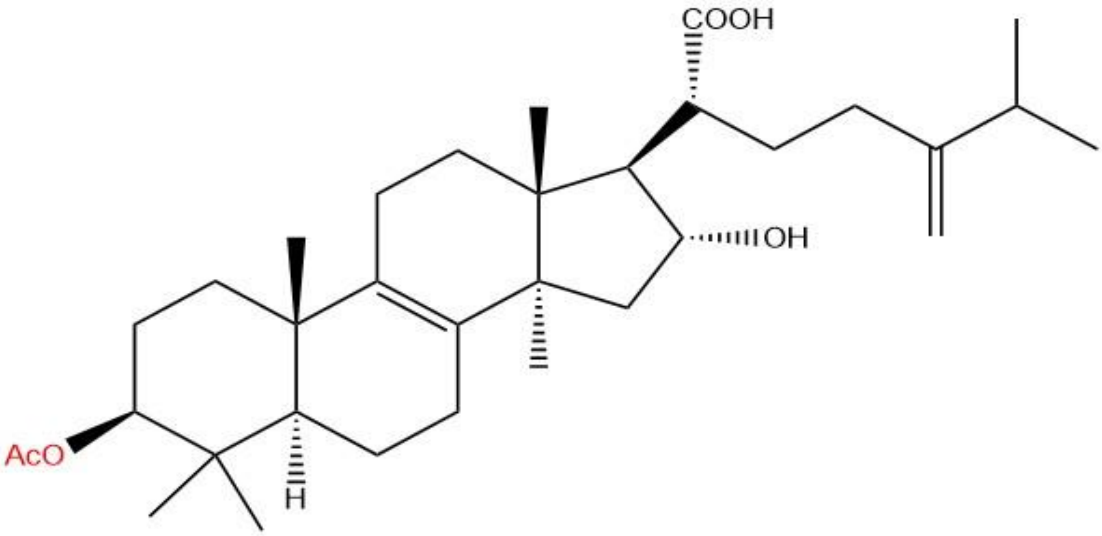

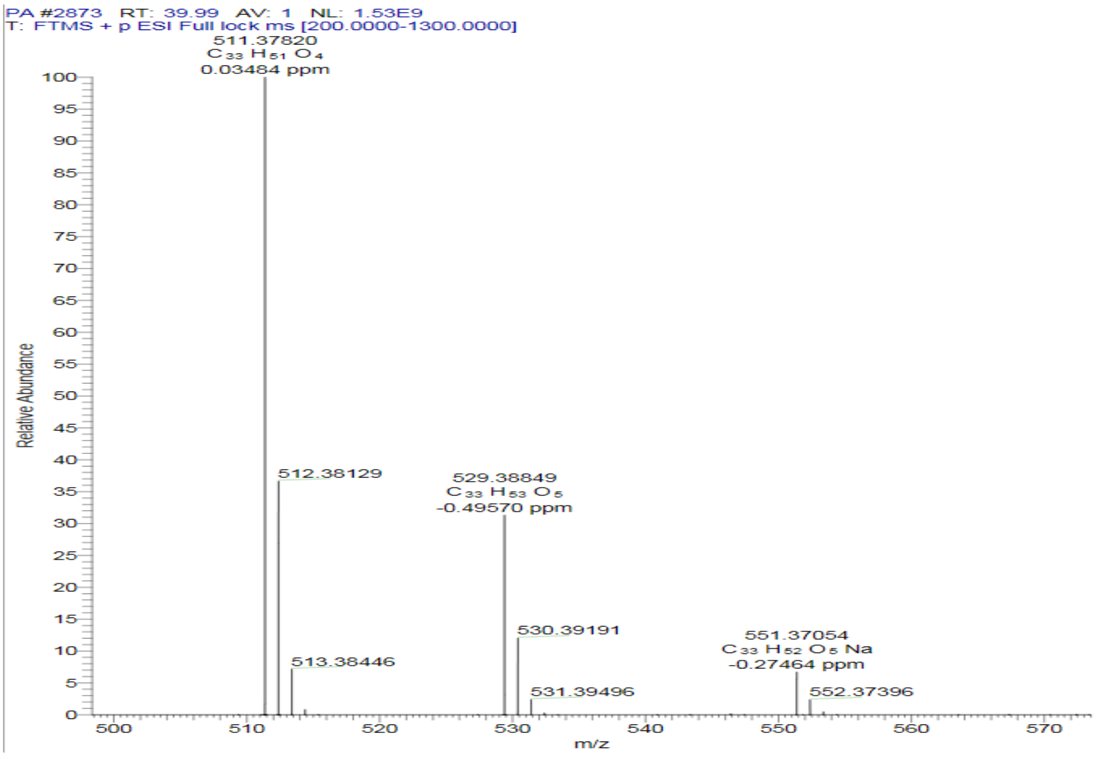

Eburicoic acid ($C_{31}H_{50}O_3$)

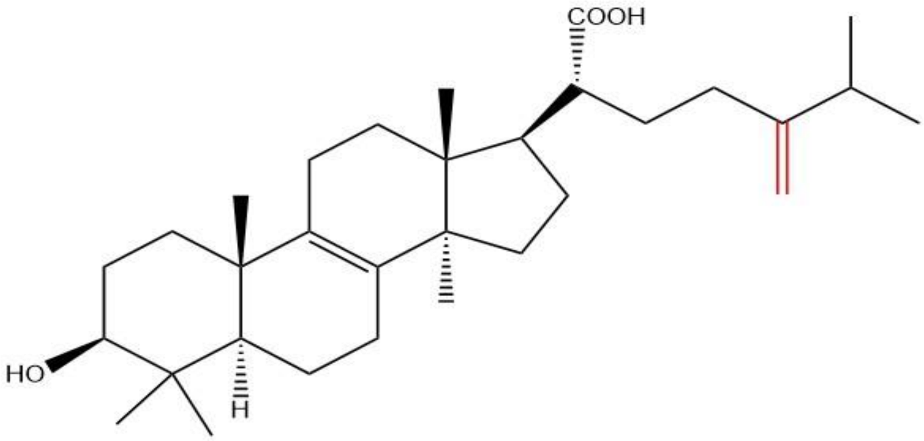

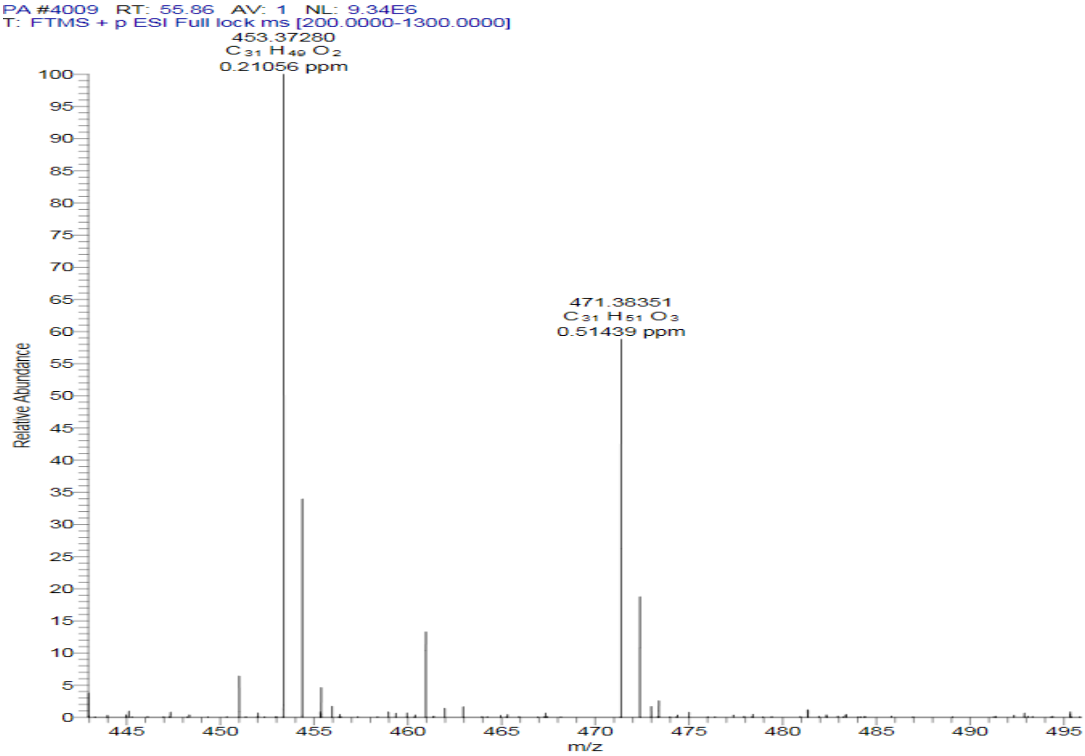

Lanosterol ($C_{30}H_{50}O$)

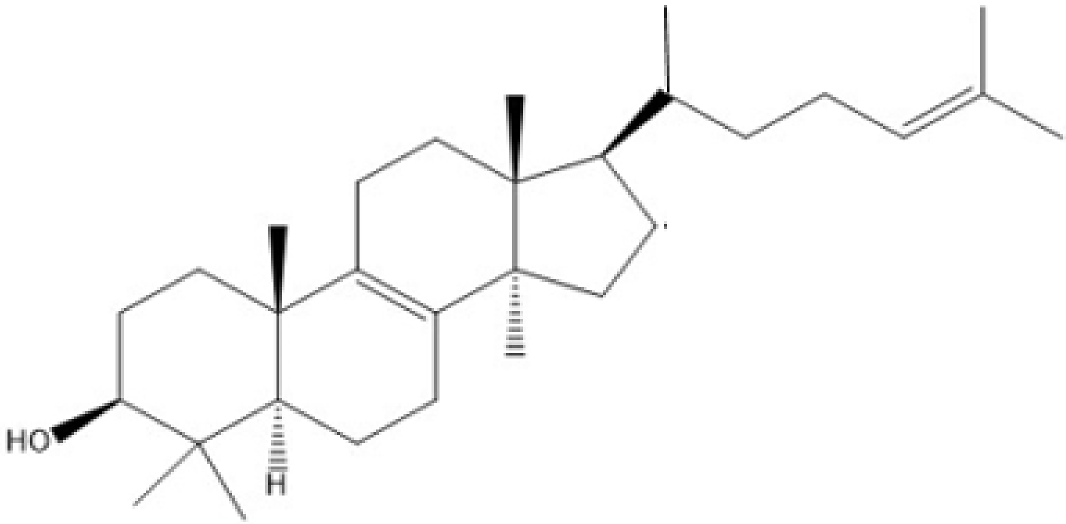

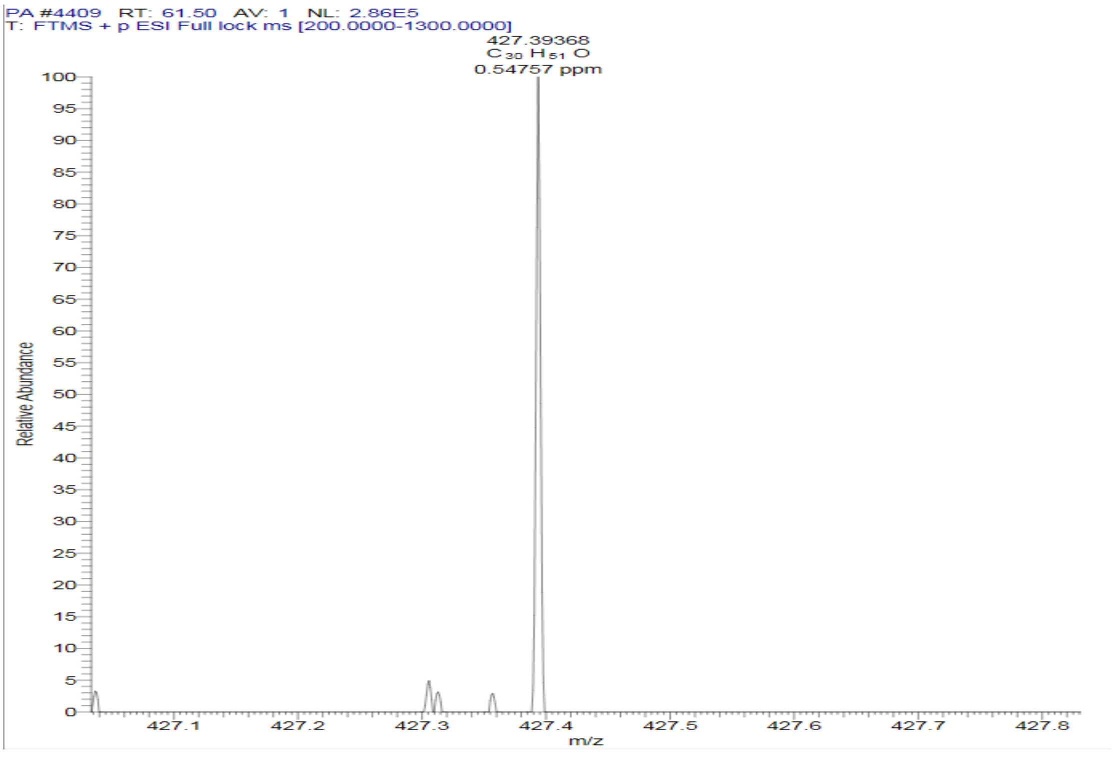

Trametenolic acid ($C_{30}H_{48}O_3$)

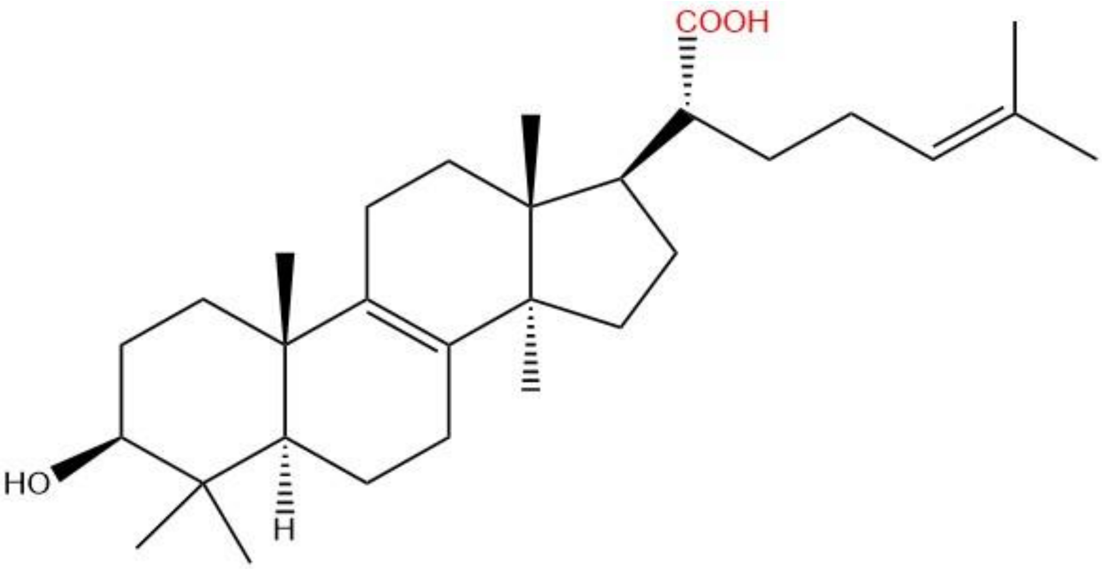

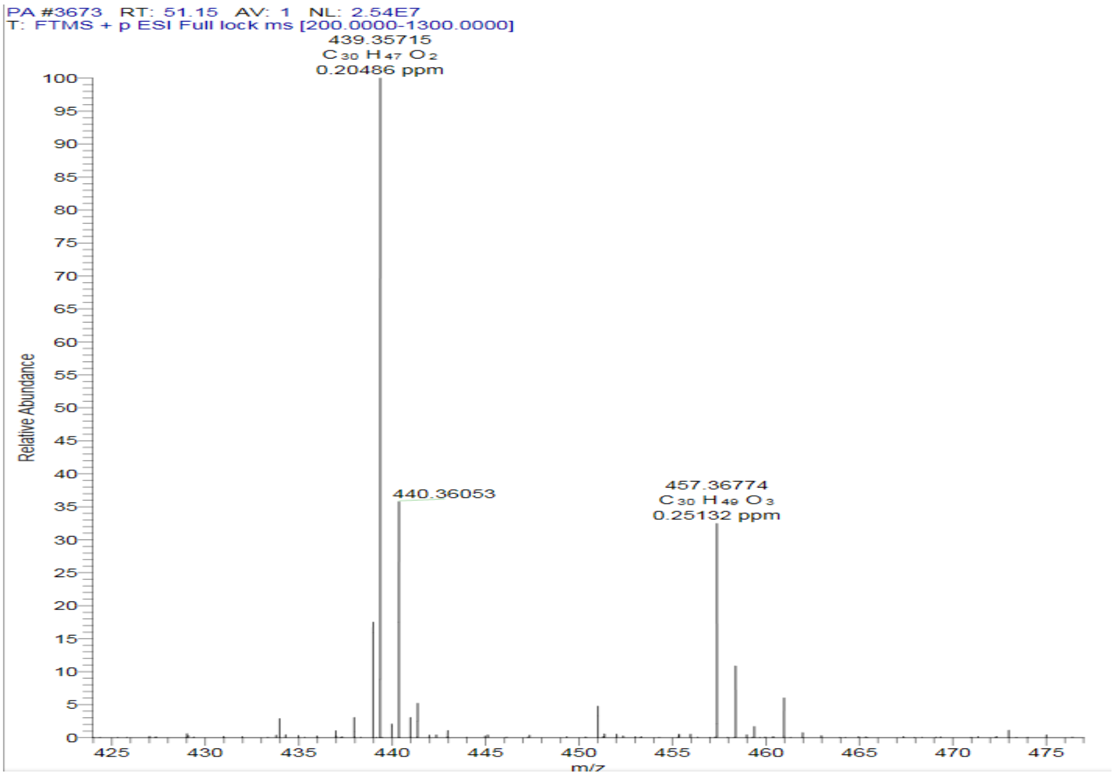

Tumulosic acid ($C_{31}H_{50}O_4$)

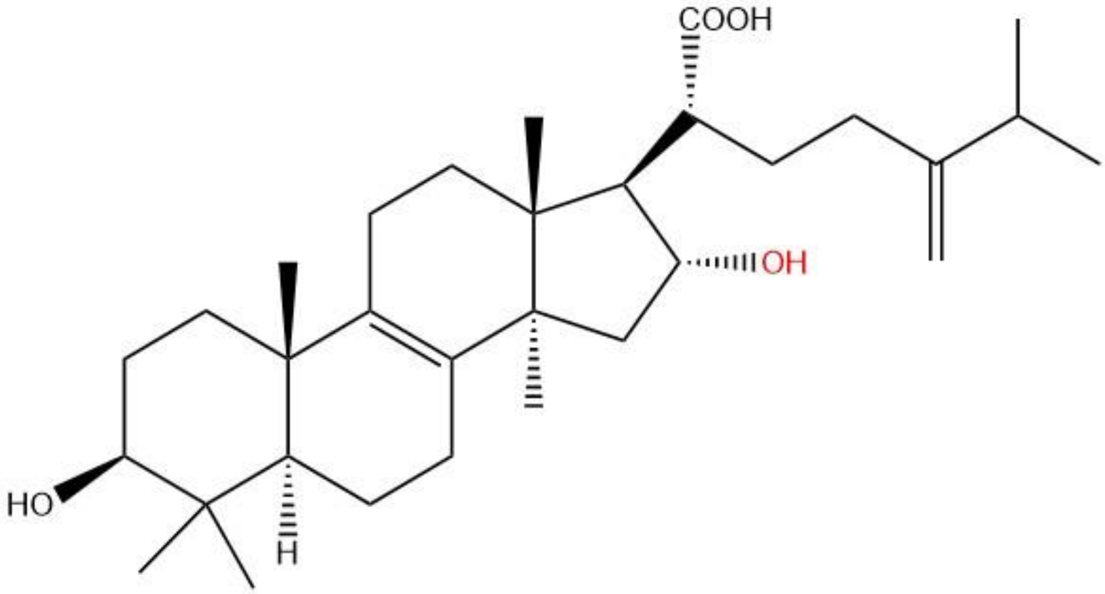

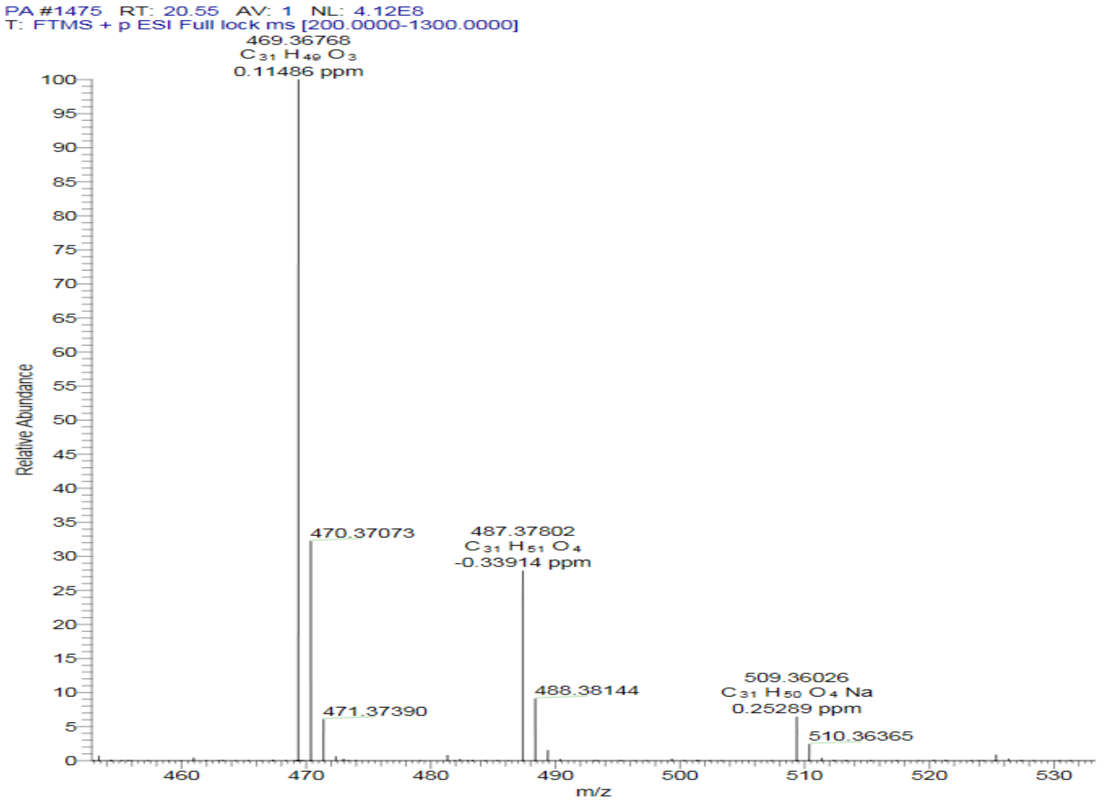

Dehydrotrametenolic acid ($C_{30}H_{46}O_3$)

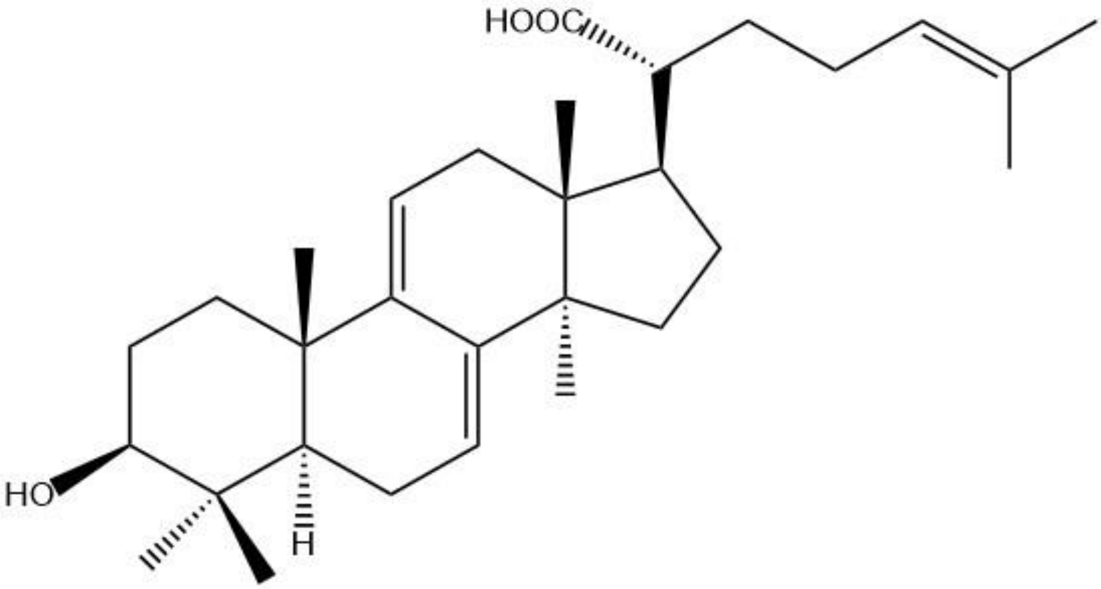

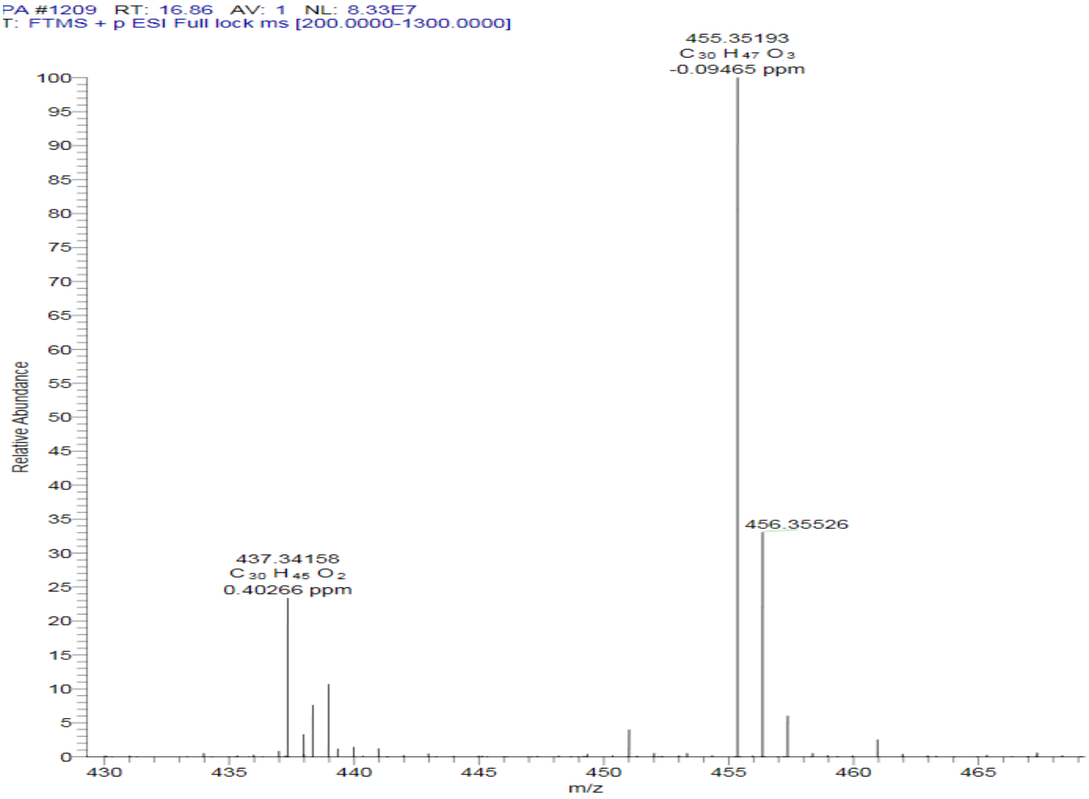

16α-Hydroxy trametenolic acid ($C_{30}H_{48}O_4$)

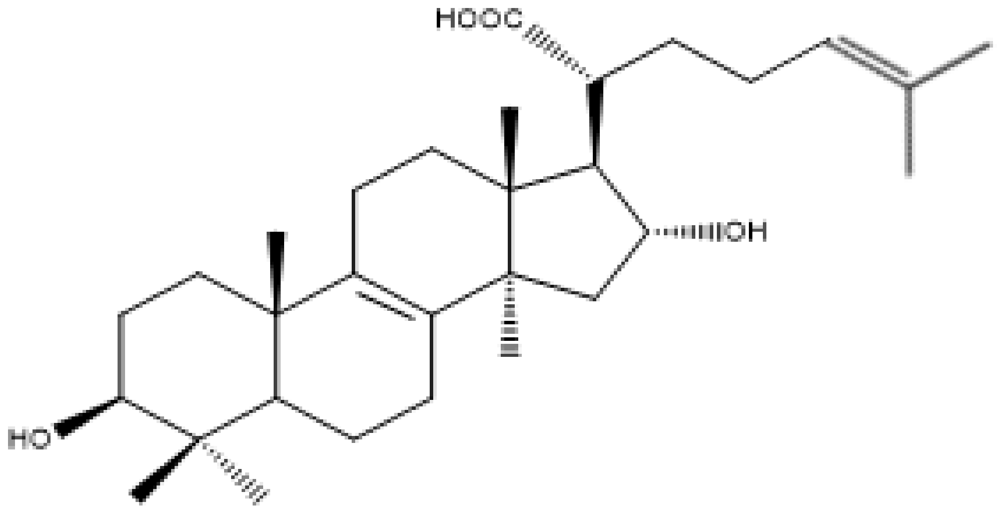

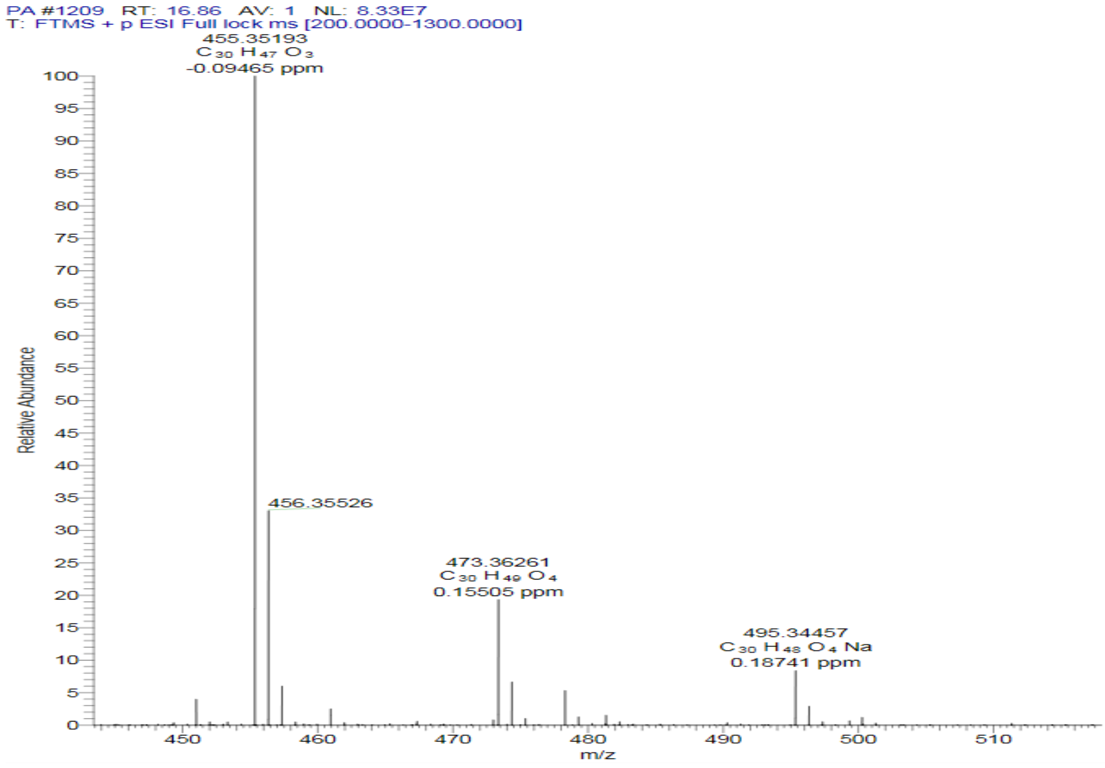

3-epi-dehydrotumulosic acid ($C_{31}H_{48}O_4$)

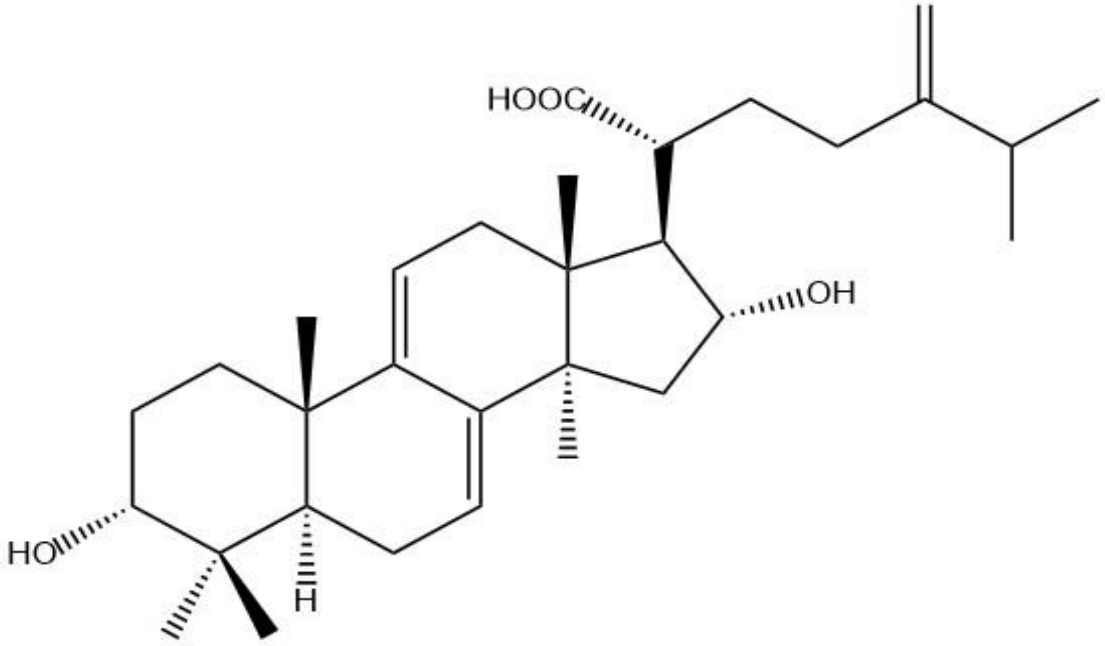

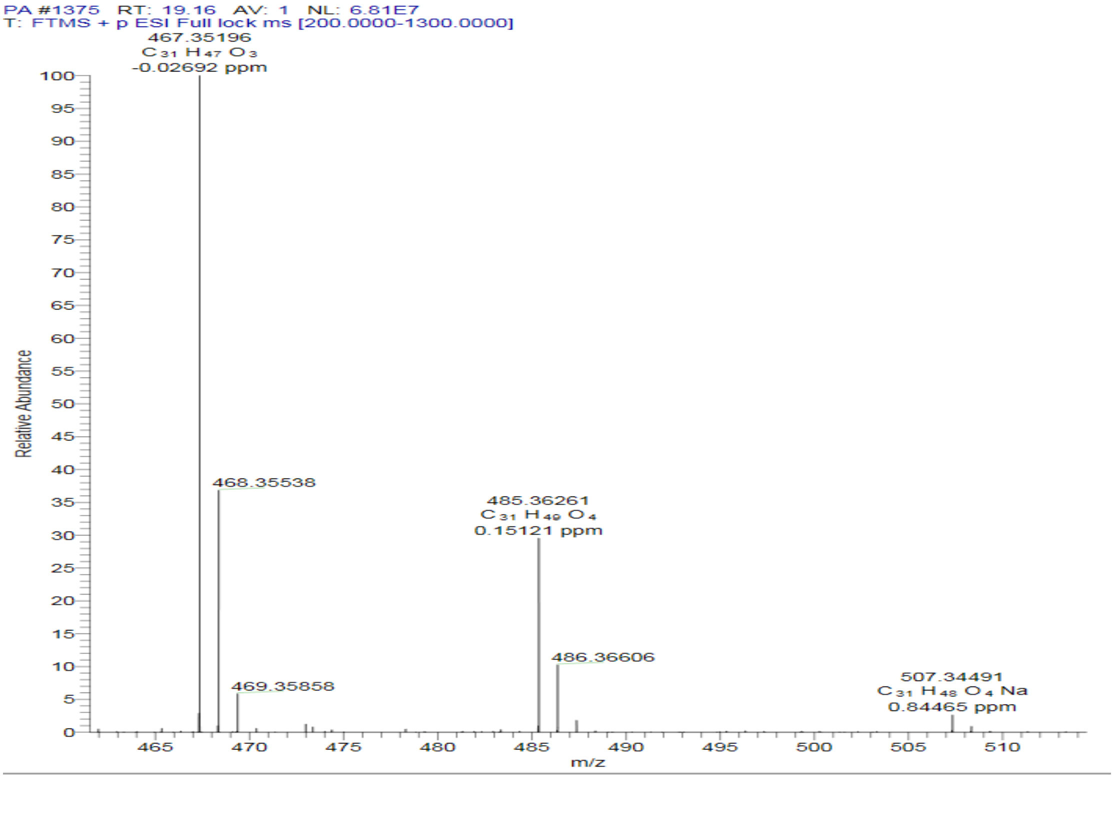

3-epi-dehydropachymic acid ($C_{33}H_{50}O_5$)

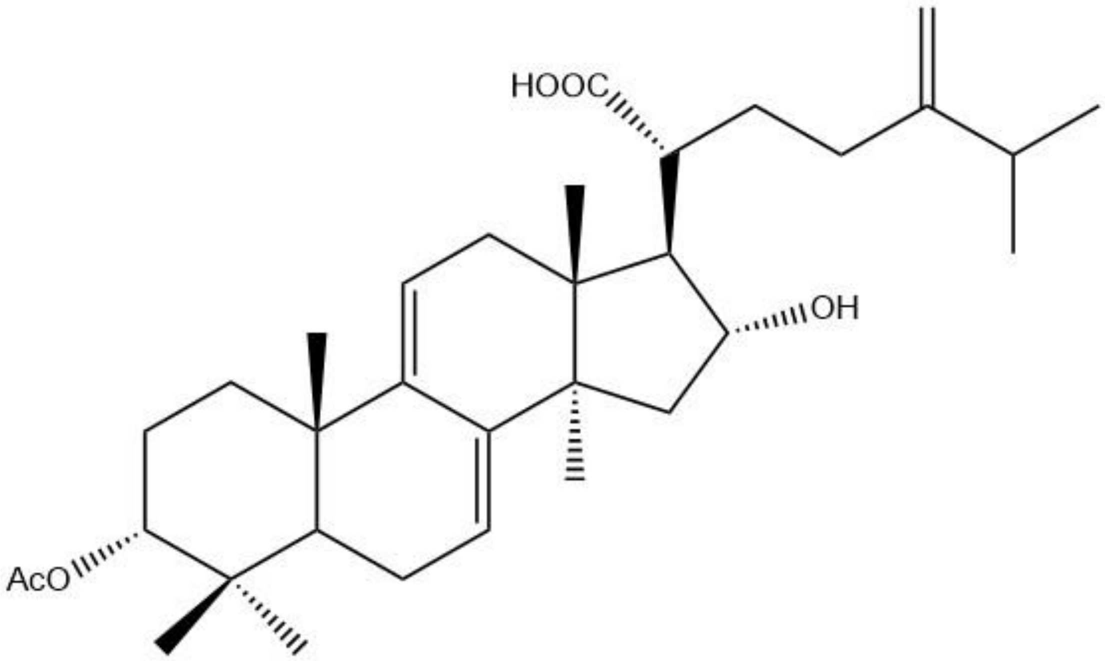

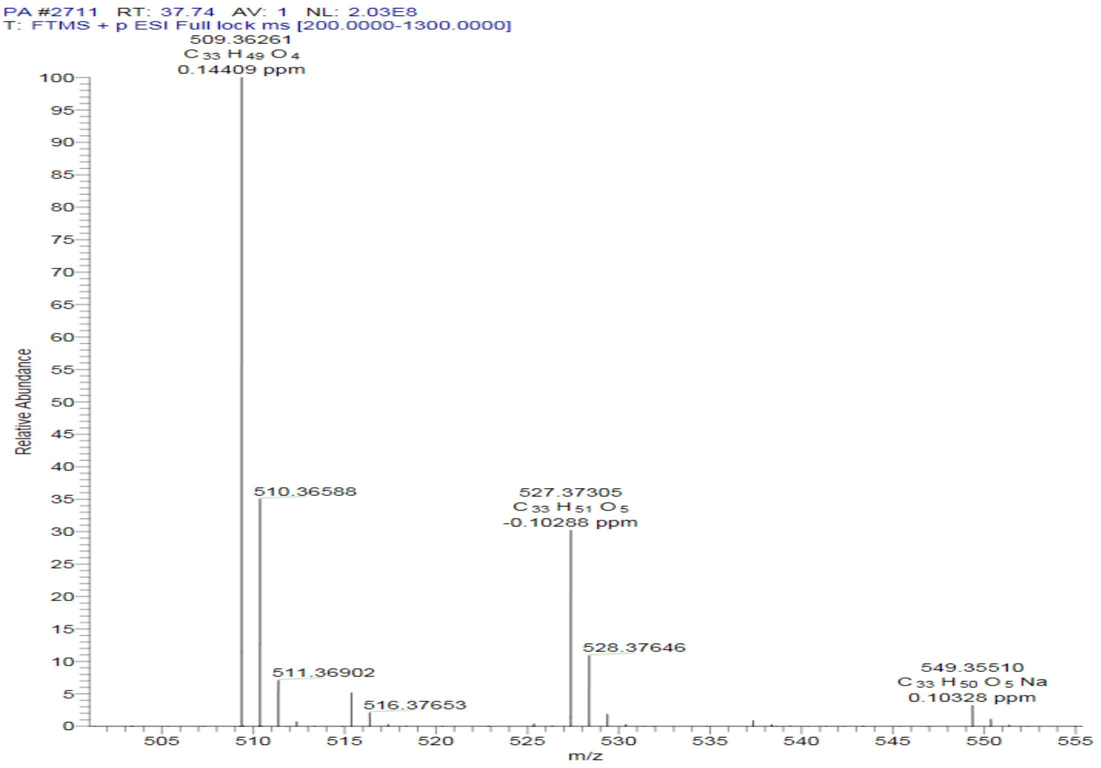

Dehydroeburicoic acid ($C_{31}H_{48}O_3$)

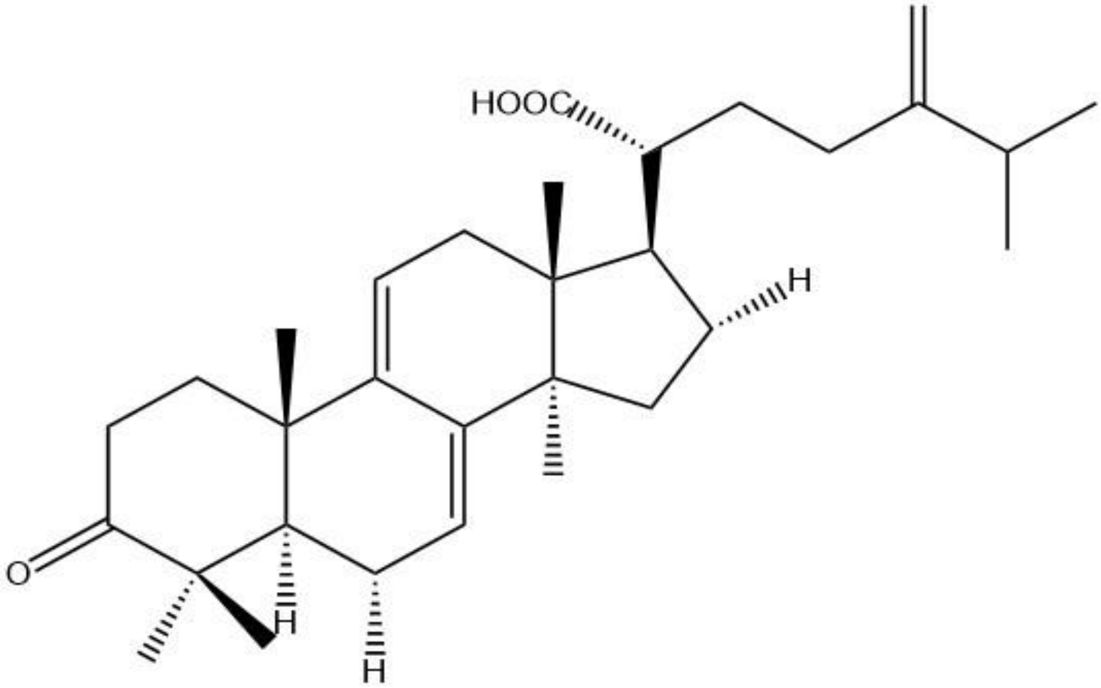

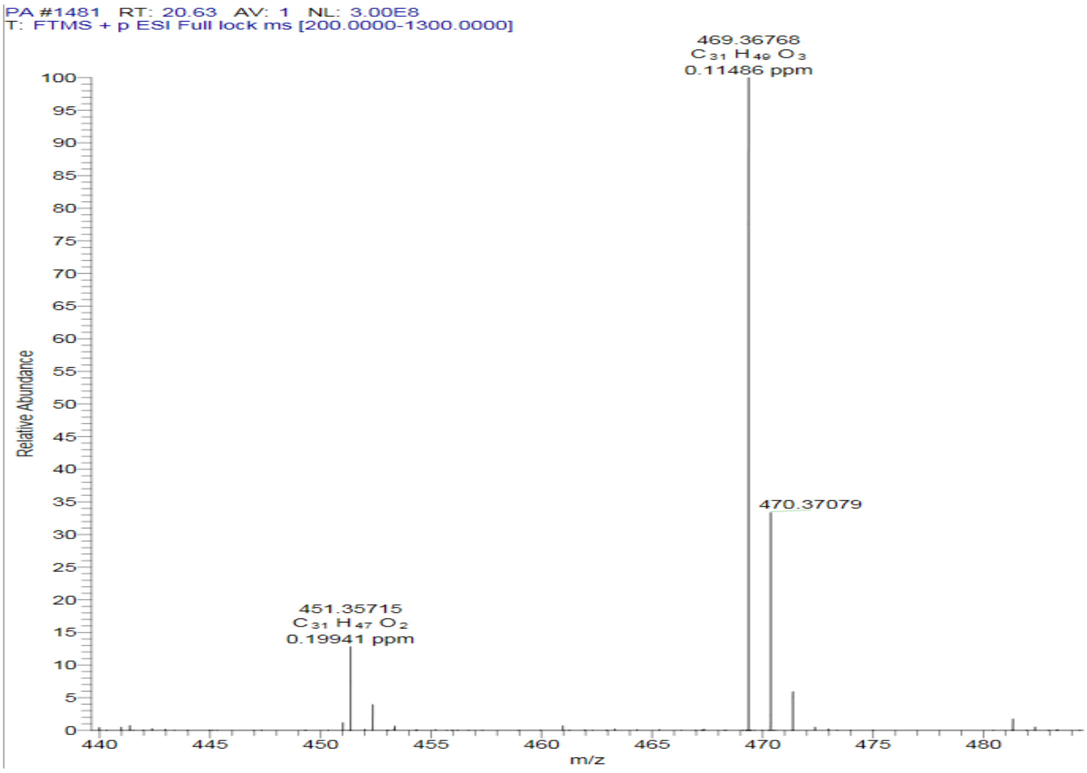

Poricoic acid C (C31H46O4)

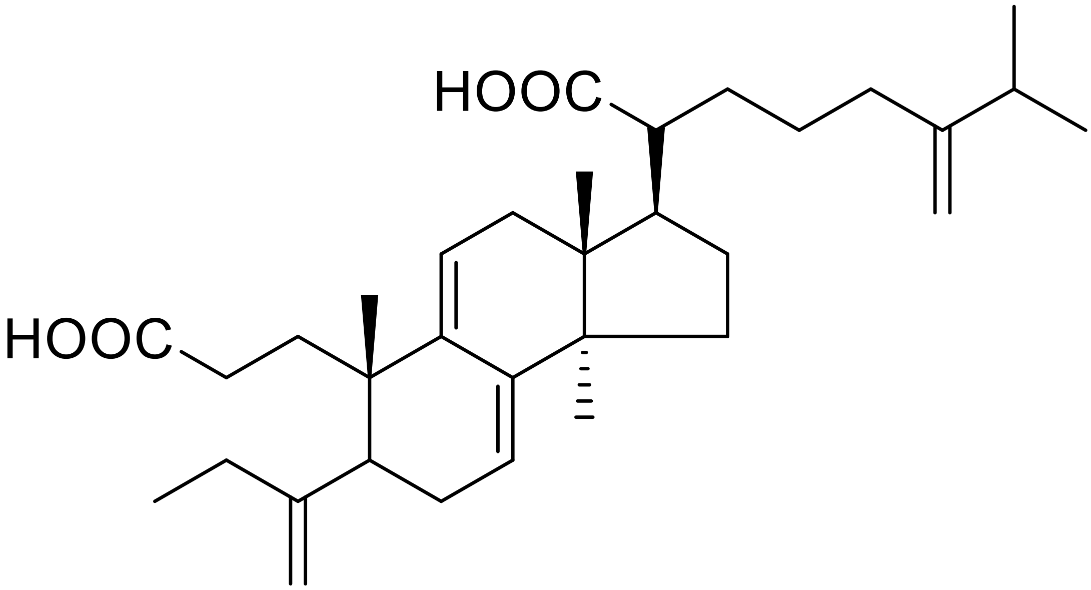

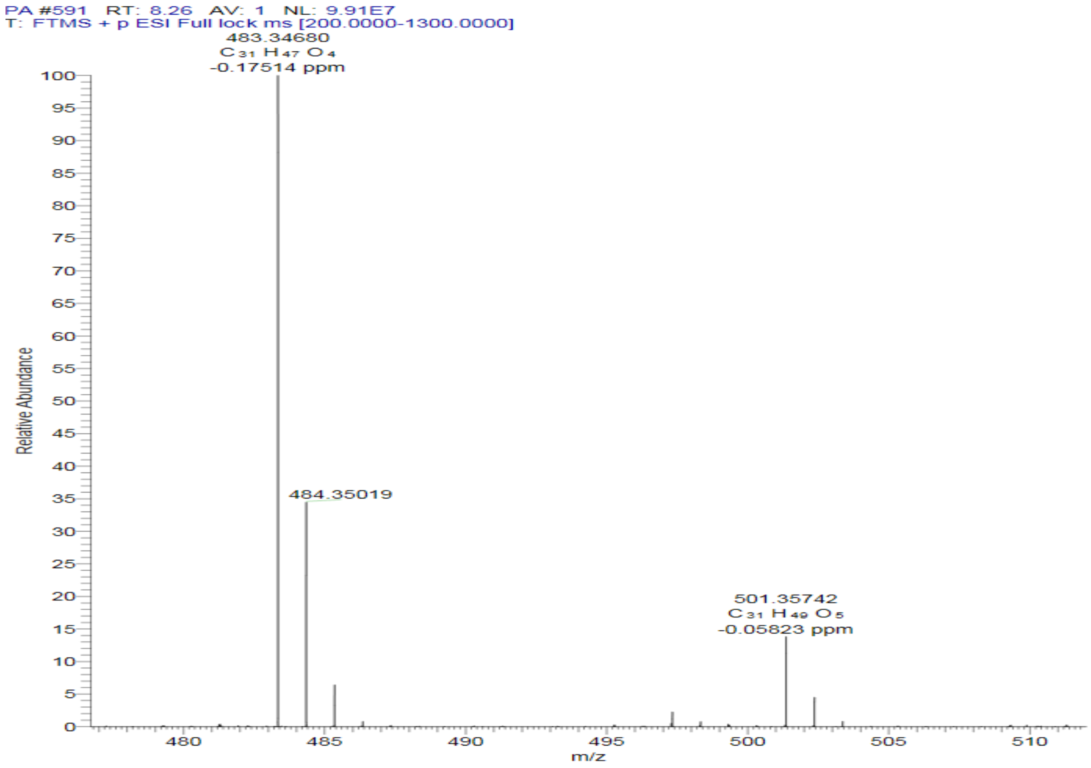

Supplement: Supplementary file 1 [file molecules-27-00143-s001.zip › Figure S1.pdf]
