# Supplementary figures and images for: Functional Analysis of Sterol O-Acyltransferase Involved in the Biosynthetic Pathway of Pachymic Acid in Wolfiporia cocos
Source: Molecules. 2021 Dec 27;27(1):143. doi: 10.3390/molecules27010143 (PMC8746942; doi:10.3390/molecules27010143)

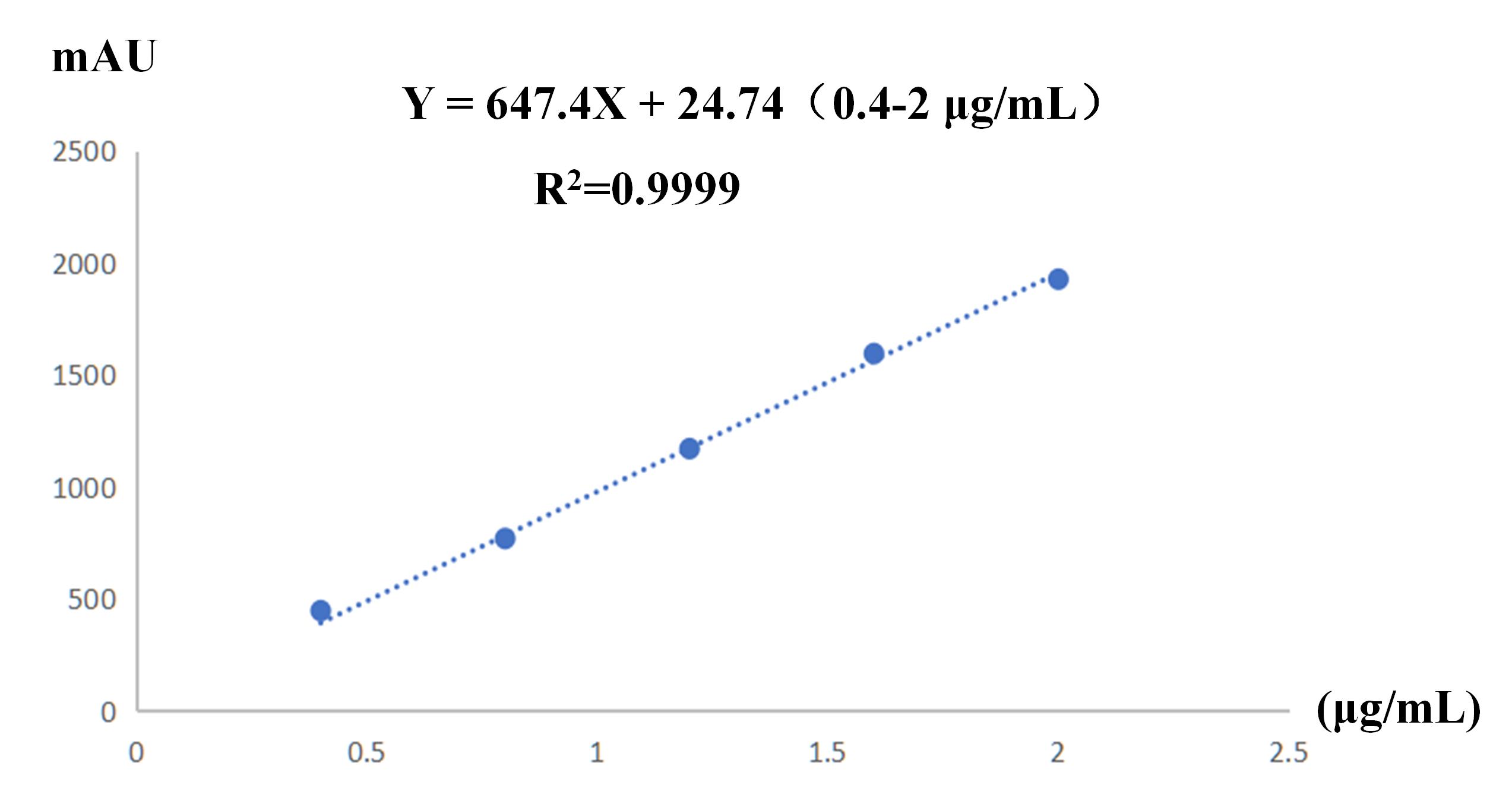

Supplement: Supplementary file 1 [file molecules-27-00143-s001.zip › Figure S2.tif]
